# Study entry, change, and death: learning from decades of follow‐up in clinical‐pathologic studie

**DOI:** 10.1002/alz.089862

**Published:** 2025-01-09

**Authors:** Ana W. Capuano, Lisa L. Barnes, Julie A. Schneider, David A. Bennett

**Affiliations:** ^1^ Rush Alzheimer’s Disease Center, Chicago, IL USA; ^2^ Rush University Medical Center, Chicago, IL USA; ^3^ Rush Alzheimer’s Disease Center, Rush University Medical Center, Chicago, IL USA; ^4^ Rush University, Chicago, IL USA

## Abstract

**Background:**

Dementia, a growing health crisis, disproportionally affects persons from racial/ethnic backgrounds and individuals with comorbidities. Latelife change in cognition is complex and nonlinear, as well as differential for these individuals. These individuals are also largely underrepresented in clinical trials. We discuss analytical methods using cognitive aging data from participants in well‐characterized clinical‐pathologic studies with long follow‐ups that may inform the design of more inclusive clinical trials.

**Method:**

We examined harmonized data from older adults enrolled in one of four ongoing clinical‐pathologic studies of cognitive aging: the Religious Orders Study (ROS); Rush Memory and Aging Project; Minority Aging Research Study and African American Clinical Core. Trajectories of cognition were ascertained with different models including Changepoint and Sigmoidal Mixed Models. Trajectories were compared by race, ethnicity, neuropathological profile, and relevant comorbidities such as insulin resistance. We studied fractions of the trajectory that could be observed in trials using composites such as the Alzheimer’s Prevention Initiative Preclinical Composite Cognitive (APCC) and the Preclinical Alzheimer’s Composite with Semantic processing (PACC5).

**Results:**

The RADC cohorts started as early as 1994 with the first (ROS) having accumulated up to three decades of follow‐up (16‐27 years for the others). Using different techniques we demonstrated that change in cognition was marked by a small retesting effect at the very beginning of the trajectory and a nonlinear accelerated decline in the years that preceded death. Using matched samples we identified similar s or rates of decline in Latinx but lower initial levels and less accelerated declines in Black older adults. We identified different smooth late‐life accelerations as a function of factors such as insulin resistance, BMI, and neuropathology. Later acceleration can start much earlier, entering segments of the trajectory potentially used in clinical trials. Trajectory characteristics (slope and variability) changed with composite, which is important in translating cohort information to the statistical planning of clinical trials.

**Conclusion:**

The examination of the trajectories with long follow‐ups using different modeling techniques can provide insights into profiles of cognitive aging. The use of these data can be one source among several to inform the design of more inclusive clinical trials.

